# Alpha Power Increase After Transcranial Alternating Current Stimulation at Alpha Frequency (α-tACS) Reflects Plastic Changes Rather Than Entrainment

**DOI:** 10.1016/j.brs.2014.12.004

**Published:** 2015

**Authors:** Alexandra Vossen, Joachim Gross, Gregor Thut

**Affiliations:** a School of Psychology, University of Glasgow, 58 Hillhead Street, Glasgow G12 8QB, United Kingdom; b Institute of Neuroscience and Psychology, University of Glasgow, 58 Hillhead Street, Glasgow G12 8QB, United Kingdom

**Keywords:** Transcranial alternating current stimulation, Alpha oscillations, Entrainment, Spike-timing dependent plasticity, Electroencephalogram, Synchronization

## Abstract

**Background:**

Periodic stimulation of occipital areas using transcranial alternating current stimulation (tACS) at alpha (*α*) frequency (8–12 Hz) enhances electroencephalographic (EEG) α-oscillation long after tACS-offset. Two mechanisms have been suggested to underlie these changes in oscillatory EEG activity: tACS-induced entrainment of brain oscillations and/or tACS-induced changes in oscillatory circuits by spike-timing dependent plasticity.

**Objective:**

We tested to what extent plasticity can account for tACS-aftereffects when controlling for entrainment “echoes.” To this end, we used a novel, intermittent tACS protocol and investigated the strength of the aftereffect as a function of phase continuity between successive tACS episodes, as well as the match between stimulation frequency and endogenous α-frequency.

**Methods:**

12 healthy participants were stimulated at around individual α-frequency for 11–15 min in four sessions using intermittent tACS or sham. Successive tACS events were either phase-continuous or phase-discontinuous, and either 3 or 8 s long. EEG α-phase and power changes were compared after and between episodes of α-tACS across conditions and against sham.

**Results:**

α-aftereffects were successfully replicated after intermittent stimulation using 8-s but not 3-s trains. These aftereffects did not reveal any of the characteristics of entrainment echoes in that they were independent of tACS phase-continuity and showed neither prolonged phase alignment nor frequency synchronization to the exact stimulation frequency.

**Conclusion:**

Our results indicate that plasticity mechanisms are sufficient to explain α-aftereffects in response to α-tACS, and inform models of tACS-induced plasticity in oscillatory circuits. Modifying brain oscillations with tACS holds promise for clinical applications in disorders involving abnormal neural synchrony.

## Highlights

• Intermittent periodic stimulation of occipital areas with alpha-tACS enhances offline EEG alpha power.
• Alpha-aftereffects cannot be explained by neuronal entrainment but are more likely due to plastic changes.
• We propose a physiological constraint to a recent model of tACS-induced spike-timing dependent plasticity.

Many human electrophysiological studies have mapped specific aspects of perception, memory, and cognition onto specific features of oscillatory brain activity, including phase and frequency (see e.g. Refs. [1–10]). Conventionally, such functional maps are established via non-invasive recording techniques such as electro/magnetoencephalography (EEG/MEG) by examining task-related modulation of oscillatory brain activity or its covariation with behavioral performance measures. However, these maps are correlational by nature and do not permit the distinction between epiphenomenal and causal functional accounts. Recent attempts to demonstrate causal roles of oscillatory brain activity in implementing function have used non-invasive brain stimulation (NIBS, [11–14]) to promote natural neural frequencies [15]. To this end, an external, periodic electromagnetic force is applied over the appropriate (potentially task-relevant) brain area at the area's preferred oscillatory frequency with the aim to synchronize the intrinsic oscillations to the external force, and to assess the associated behavioral consequences (reviewed in [9,16,17]). A promising NIBS-approach for such controlled intervention is transcranial alternating current stimulation (tACS). tACS involves the induction of a weak sinusoidal electric current between two or more scalp electrodes [17,18] which can be applied at biologically relevant frequencies (i.e. those frequencies spontaneously exhibited by neural networks). In line with the view that tACS selectively interacts with underlying brain oscillations and functions, increasing numbers of behavioral studies show that frequency-tuned tACS affects specific aspects of perception [19–25], memory [26–28], motor function [29–34] and higher-order cognition [35–37], in many instances matching the known correlational links between EEG/MEG-frequency and function [19–36] (but see Refs. [38–41]). However, little is known about the electrophysiological underpinnings and the precise mechanism through which tACS procures its effects.

Two main hypotheses have been suggested: that tACS directly entrains underlying brain oscillations [17,18,42,43] and/or that tACS leads to synaptic changes via spike-timing dependent plasticity mechanisms [28,44]. Entrainment of brain oscillations refers to the temporal alignment of intrinsic brain activity to periodic (e.g. sensory, electrical, or magnetic) stimulation [16]. It involves i) a neural population capable of producing rhythmic activity at the desired frequency, and ii) phase alignment of this intrinsic activity to the phase of the external driving source. tACS-induced entrainment has been demonstrated during (online to) tACS both behaviorally [24,30,37] and electrophysiologically in humans [20,37], as well as in animal studies both in vitro and in vivo [45–49]. The latter work, as well as research on photic driving in humans [50,51], indicate that entrainment is strongest when stimulation frequency is at or close to the network's preferred frequency (eigenfrequency). Specifically, the stimulated system is then expected to respond at the driving frequency rather than its eigenfrequency [45,52]. Spike-timing dependent plasticity, on the other hand, has been suggested to underlie the enhancement of oscillatory brain activity at tACS-frequency *beyond stimulation* (i.e. *offline* to tACS) [28,44]. Such aftereffects have been reported in the form of enhanced posterior α-power after prolonged (ca. 10–20 min) occipito-parietal α-tACS (or α-tACS with a DC-offset) [20,24,44] that lasted for at least 30 min [53].

In the present study, we tested to what extent plasticity can account for tACS-aftereffects when controlling for entrainment “echoes,” i.e. entrained activity that remains stable after the end of rhythmic stimulation. To this end, we employed an intermittent tACS-protocol and applied short parieto-occipital α-tACS trains interrupted by breaks of equal duration. Total tACS-duration was comparable to the continuous α-tACS-protocols previously reported to lead to offline α-enhancement [20,24,44,53]. In order to assess the contribution of entrainment echoes to the α-aftereffect, we manipulated phase-continuity (continuous versus discontinuous) between successive α-tACS trains. Based on observations online to tACS (see Ref. [20]) as well as theoretical groundwork [44,52], we reasoned that if entrainment echoes come into play, α-enhancement should be 1) stronger when intermittent α-tACS trains are applied in phase-continuous versus phase-discontinuous regimes, 2) centered at stimulation frequency rather than intrinsic eigenfrequency, and 3) stronger when the stimulation frequency matches the spontaneous α-frequency, while 4) EEG phase-locking to the phase of the tACS-train should outlast tACS-offset as a minimum requirement for stable entrainment over minutes. Our EEG results confirmed enhanced α-power after α-tACS compared to sham stimulation, but did not reveal any of the hypothesized offline entrainment characteristics. Consistent with plasticity as the predominant cause for aftereffects, α-enhancement 1) occurred irrespective of phase-continuity between trains, 2) was observed at spontaneous α-peak frequency, and was 3) neither stronger with tACS at intrinsic α-frequency, nor 4) associated with prolonged phase-locking beyond tACS.

## Materials and methods

### Participants

12 healthy volunteers (6 male, age 27 ± 5 years) completed this study. All volunteers gave written informed consent and received monetary compensation for their participation. The study was approved by the local ethics committee of the College of Science and Engineering, University of Glasgow. No participants reported a history of neurological/psychiatric disorders or any other contraindication to tACS (current use of psychoactive medication/drugs, metal implants, pregnancy).

### tACS

**Figure 1:**
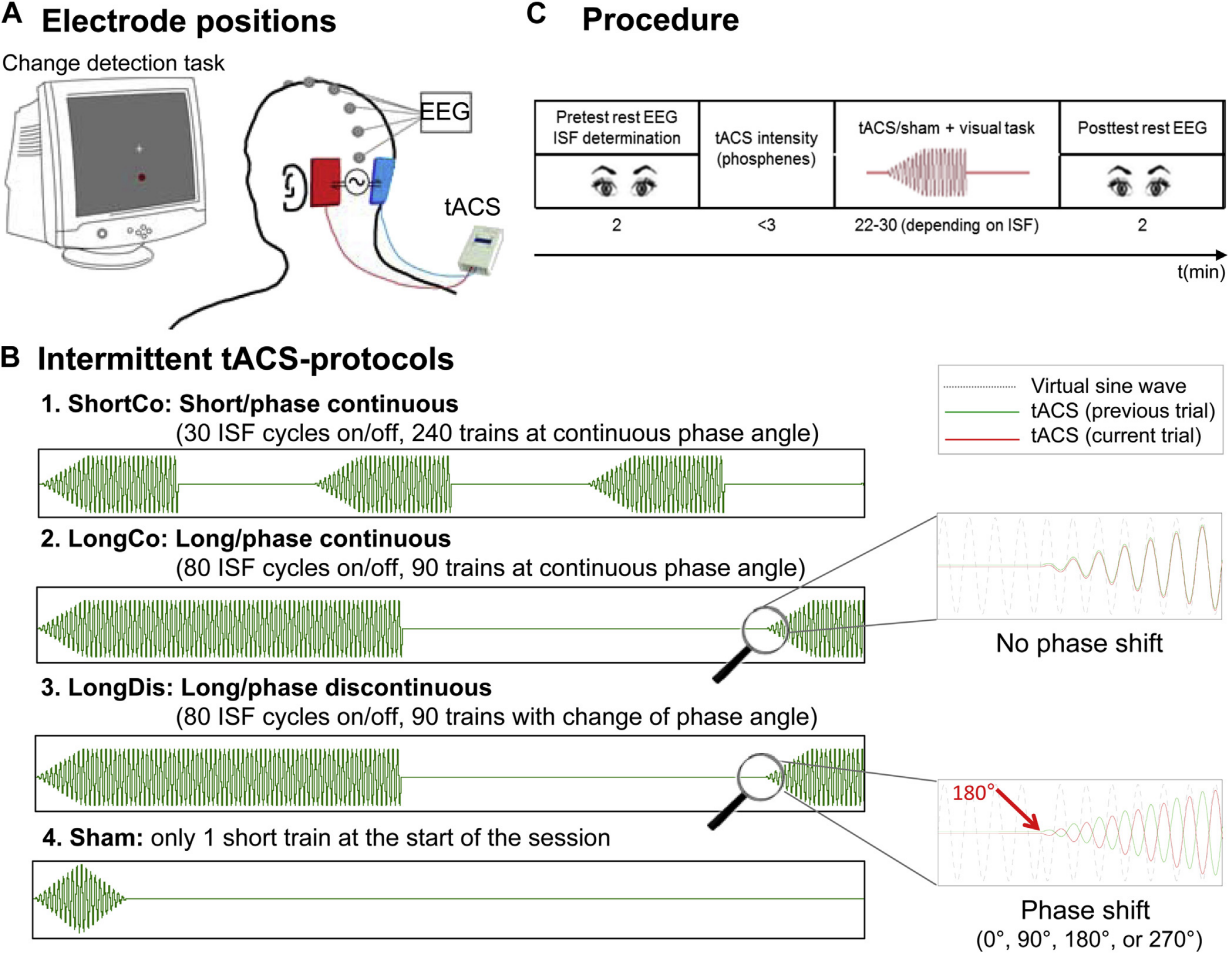
Experimental design. A) Experimental setup and C) procedure. For details refer to section Procedure. B) Examples for the different tACS protocols. For details refer to section tACS.

tACS was administered through a battery driven constant current stimulator (DC Stimulator Plus, NeuroConn, Ilmenau/Germany) controlled through Spike2 software via a Power1401 mkII microcomputer (both Cambridge Electronic Design, Cambridge/UK). 5 × 7 cm^2^ rubber electrodes in saline-soaked sponges (0.9%-NaCl) with a thin layer of electrode gel were attached to the scalp with rubber bands. Electrodes were placed bilaterally over PO7/PO9 and PO8/PO10 of the 10/10-system (Fig. 1A; cf. [44]).

Individual stimulation frequency (ISF) and intensity were determined once, in the first session, for all four sessions. ISF was determined from resting EEG with eyes open by identifying each individual peak frequency in the α-range (8–12 Hz) at electrode POz using Fast Fourier Transforms (FFTs, frequency resolution .5 Hz) and ranged from 8 to 11 Hz across participants. tACS-intensity was adjusted below individual phosphene- and discomfort threshold using a staircase procedure (see Supplementary Information) and ranged from 1.35 to 2 mA (peak-to-peak).

tACS was administered in a within-subject design with three active conditions and one sham condition (Fig. 1B) on four different days. In all active conditions α-tACS at ISF was applied in an intermittent on/off pattern. Total stimulation duration (amount of on-time) in each active condition was constant for any particular participant (7200 α-cycles at ISF) but varied across participants due to the variability in individual posterior α-frequency (i.e. from ∼11 min for an ISF of 11 Hz to ∼15 min for an ISF of 8 Hz). Total session duration was twice the length of total stimulation time (or equivalent for sham). Across conditions, we varied the length of single tACS-epochs (on-period) as well as phase-consistency across epochs. In the *short, phase-continuous condition* (*ShortCo*) (Fig. 1B.1), tACS was switched on for 30 cycles (i.e. on-periods of 3 s in participants with a 10 HzISF) followed by an off-period of the same duration. This was repeated 240 times with phase continuity between successive on-states (i.e. by adjusting amplitude, but not phase, of a virtual sine-wave spanning the whole stimulation session). In the *long, phase-continuous condition* (*LongCo*) (Fig. 1B.2), tACS was switched on/off with phase continuity (as above) for 80 cycles (i.e. on/off for 8 sepochs in participants with a 10 HzISF) in 90 repetitions. The *long, phase-discontinuous condition* (*LongDis*) (Fig. 1B.3) was identical to LongCo, except that phase-continuity was disrupted across single tACS-epochs by introducing a phase shift of 0°, 90°, 180°, or 270° to the virtual sine wave during off-periods (approximately equal probability) with respect to the previous on-period, thus initiating tACS at a different phase angle. In all active conditions, tACS-intensity was ramped up over the first 10 cycles to minimize unpleasant sensations under the electrodes. Finally, in the *sham condition*, only one short tACS-train (10 cycles ramp-up, 10 cycles ramp-down) was administered at the beginning of the session. This condition was included to control for tACS-unspecific effects (e.g. fatigue).

### EEG recordings

EEG was recorded at the midline sites Fpz, Fz, Cz, CPz, Pz, and POz (referenced to AFz) (Fig. 1A; cf. [44]) using a BrainAmp MRPlus amplifier (BrainProducts, Munich/Germany). Vertical eye movements were recorded from two additional electrodes above and below the right eye. The signal was bandpass-filtered online between 0.1 and 1000 Hz and digitized at a sampling rate of 5 kHz.

### Procedure

Each participant underwent four sessions of maximally 2 h each. Sessions were at least 3 days apart. Preparation of tACS- and EEG-electrodes took ∼45 min. Data acquisition started with 2 min of resting EEG with eyes open (pre-test) (Fig. 1C). Participants then underwent one of the stimulation protocols in counterbalanced order while EEG was continuously measured. For the duration of each protocol, participants performed a visual vigilance task to maintain alertness (see Supplemental Material). Finally, an additional 2 min of resting EEG with eyes open was recorded (post-test).

### EEG analysis

Analyses were conducted in BrainVision Analyzer 2.0 (BrainProducts) and Matlab (MathWorks, Natick/USA) using the Fieldtrip Toolbox (Donders Centre for Cognitive Neuroimaging, Nijmegen/NL). For all statistical analyses, non-parametric tests were used (IBM SPSS Statistics, version 19.0, IBM Corp, Armonk/US; see Supplemental Material). The reported results refer to the signal recorded at electrode POz except for one subject, where due to excessive noise in one condition we chose to analyze Pz instead.

#### Analysis of aftereffects in α-power (pre vs post-test)

The analysis of the pre- and post-tACS EEG measurements largely followed [24,44]. The “eyes open” resting EEGs were segmented into 1 s-epochs. Epochs containing eye movement and muscle contraction artefacts were discarded. A fast Fourier transform (FFT) for frequencies between 1 and 20 Hz (.5 Hz resolution) was calculated for individual epochs using a Hanning window and 2 s zero-padding. The resulting spectra of each condition were averaged across epochs as well as across the individually determined α-bands (ISF ± 2 Hz) per tACS-condition. Normalized relative changes of mean α-power from pre-test to post-test were calculated in decibel (*change* = 10^∗^ log10 (*post-test*/*pre-test*)).

#### Analysis of offline changes in α-activity in the intermittent, tACS-free intervals

##### Pre-processing

Epochs of 2.3 s duration were extracted from the EEG between successive tACS-trains starting 100 ms after tACS-offset (due to residual tACS-artefact in the first 100 ms of EEG). Very noisy epochs and epochs with eye blinks at trial-onset were removed after visual inspection of the data. Remaining eye blink contaminations were then eliminated (1) using a principal component denoising approach (implemented in Fieldtrip) with the bipolar EOG-derivation as reference signal (using 1–8 Hz bandpass-filtered data to optimize blink detection, and applying the respective PCA-weights to the original data), and (2) by discarding the epoch if elimination was not successful. Because both long conditions had significantly lower trial numbers than sham and ShortCo, we randomly sampled (without replacement), for each participant and each condition, as many trials as available in the condition with the lowest trial number. All subsequent analyses were conducted on these subsamples of equal size.

##### Analysis of relative change in induced α-power

We followed a similar pipeline as for the analysis of the pre- and post-tACS data. From the pre-processed data, two 1 s-epochs were cut at the beginning of each 2.3 s-interval. These were divided into blocks of early and late epochs, respectively (i.e. first and second half of the experimental session). FFT-spectra were calculated for each 1 s-epoch separately, and subsequently averaged per block and tACS-condition. Average power in the individual stimulation band (ISF ± 2 Hz) for each block was again log-normalized to pre-test power.

##### Analysis of α phase-locking

To obtain phase information, pre-processed data were bandpass-filtered in individual α-bands (ISF ± 2 Hz) and Hilbert-transformed. The resulting complex values were normalized to unit amplitude. The phase locking value (PLV) was computed for each time point as the absolute value of the mean of these normalized complex values across trials. PLVs were averaged across the first 200 ms of the 2.3 s-epoch (i.e. from 100 to 300 ms post artefact) and then across epochs within early and late blocks in each tACS-condition.

## Results

### α-Aftereffect replicated with intermittent α-tACS

**Figure 2:**
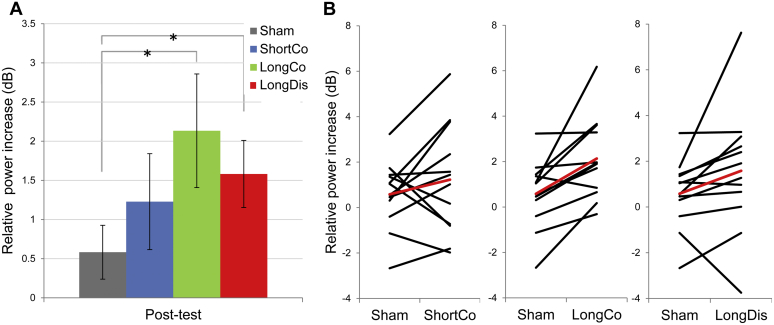
Alpha-aftereffects across protocols. A) Mean relative increase (dB) in individual alpha band power from pre-test to post-test. Both long protocols are followed by a significantly higher alpha-increase compared to sham. Asterisks reflect significant pairwise comparisons using Wilcoxon Signed Rank Tests (*α* = 0.05). Only the respective comparisons between Sham and LongCo (lower brace), and Sham and LongDis (upper brace), were significant. B) Relative increase in mean power in the individual alpha band (individual stimulation frequency (ISF) ± 2 Hz) from pre-test to post-test per participant. Each active stimulation condition is compared to Sham. Black lines represent individual differences between sham and active conditions, red line represents the mean difference. Most volunteers show a greater increase after stimulation with long (80 cycles at ISF) trains compared to sham.

We found α-power (ISF ± 2) to be enhanced after intermittent α-tACS (pre versus post-test), with participants showing on average stronger α-enhancement after active tACS as compared to sham (see Fig. 2A for group-averages, Fig. 2B for individual data). Specifically, in both long conditions individual responses were highly consistent across participants, with 11 out of 12 participants showing stronger α-enhancement to α-tACS in the long phase-continuous and 10 out of 12 in the long phase-discontinuous condition as compared to sham (Fig. 2B, middle and right panel: LongCo vs. Sham and LongDis vs Sham; Suppl. Fig. 1, right). Statistically, a main effect of condition was confirmed by a Friedman Test (*Χ*^2^_3_ = 11.1, *P* = 0.011). Breaking down this effect using the Wilcoxon Signed Rank Tests (2-tailed) indeed revealed significant α-enhancement only for both long tACS conditions compared to sham (LongCo vs. Sham: *Z* = 2.82, *P* = 0.005; LongDis vs. Sham: *Z* = 2.04, *P* = 0.041; ShortCo vs. Sham: *Z* = 1.26, *P* = 0.21), replicating the α-aftereffect previously reported for continuous α-tACS-protocols [20,24,44,53].

### α-Aftereffect does not differ between phase-continuous and phase-discontinuous protocols

α-enhancement after active tACS (LongCo > LongDis > ShortCo) did not significantly differ between conditions (all *P* > 0.05, Fig. 2A). While long intermittent tACS significantly enhanced α-power (relative to sham), this enhancement was observed irrespective of phase-continuity between tACS-trains. Hence, introducing phase jitter during tACS did not disrupt the α-aftereffect, which speaks against prolonged entrainment echoes contributing to the aftereffects.

### α-Aftereffects do not peak at stimulation frequency, but at preferred cortical frequency

**Figure 3:**
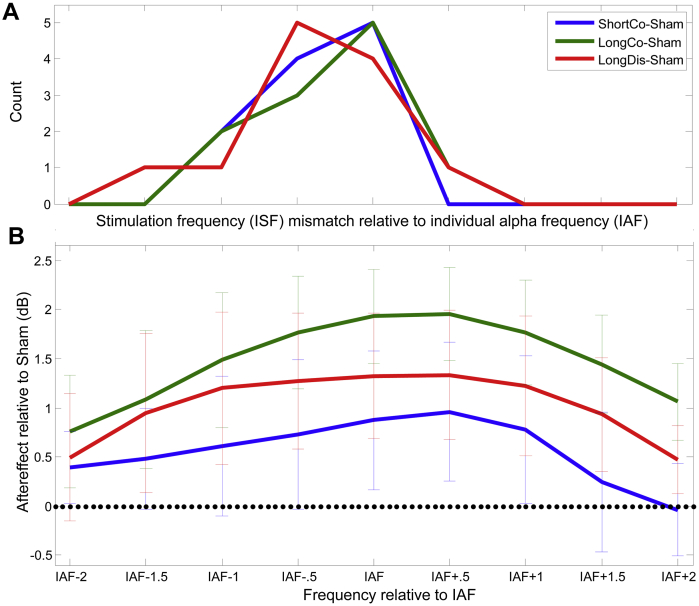
Alpha-aftereffects relative to IAF and ISF. A) Individual stimulation frequency (ISF) relative to individual alpha frequency (IAF). The distribution shows that there was a tendency to stimulate at a lower frequency than the “optimal” alpha frequency. B) IAF-aligned alpha aftereffects (difference between active protocols and sham) in mean relative power increase from pre-test to post-test (dB). Frequencies within the individual alpha band are defined by the IAF measured on the day of each session. The average increase tended to be stronger at IAF and above, i.e. slightly higher than at ISF. Error bars represent standard error of the mean.

While we stimulated at a fixed frequency (ISF = individual α frequency (IAF) at day 1), several participants showed variable IAF across sessions. This was established by randomly sampling (1000 repetitions with replacement) and averaging subsets of spectra from 1 s epochs in pretest-EEG within each session to extract peak-frequency in the 8–12 Hz-range. IAF on a given day was defined as the mode of these peaks. As a consequence, ISF deviated from IAF between sessions for several participants (range: −1.5 Hz to +3.0 Hz). This allowed us to assess whether aftereffects peaked at ISF or spontaneous IAF. Note that ISF was in most cases slightly below the IAF of a given session (Fig. 3A). Breaking down the α-band into nine bins (IAF−2 to IAF+2, in 0.5 Hz steps) (Fig. 3B), we found that tACS-aftereffects (LongCo > LongDis > ShortCo) peaked at IAF and IAF+0.5 Hz (rather than ISF), i.e. not showing the left-skew of the ISF histogram (see Fig. 3B). Separate Friedman Tests on the relative α-increase in the IAF-centred α-band (IAF−0.5 Hz to IAF+0.5 Hz) and the two flanker α-bands (IAF−2 Hz to IAF−1 Hz/IAF+1 Hz to ISF+2 Hz) revealed significant aftereffects in the IAF-centred band (*Χ*^2^_3_ = 8.1, *P* = 0.044) and the higher α-band (*Χ*^2^_3_ = 9.0, *P* = 0.029). At the IAF-centred band, the contrasts of both LongCo- and LongDis-conditions against Sham were significant (Wilcoxon Signed Rank Test; LongCo: *Z* = 2.90, *P* = 0.004; LongDis: *Z* = 1.96, *P* = 0.05; all other *P* > 0.05). In the higher α-band, only LongCo was different from Sham (*Z* = 2.51, *P* = 0.012). Importantly, repeating the same analysis but now centred on ISF (instead of IAF) did not reveal significant tACS-related α-aftereffects at ISF (ISF−0.5 Hz to ISF+0.5 Hz, Friedman *P* > 0.05). Hence, tACS-induced aftereffects were observed at or above the preferred cortical frequency but not at stimulation frequency, which again is inconsistent with prolonged entrainment echoes contributing to the aftereffect.

### No enhancement of α-aftereffects when stimulation and preferred frequency match

**Figure 4:**
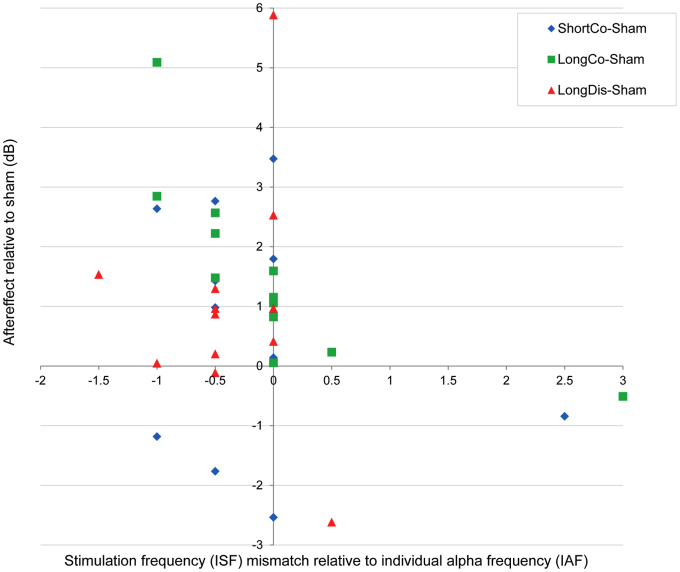
Correlations between relative alpha increase and extent of the mismatch between individual stimulation frequency (ISF) and individual alpha frequency (IAF). Data points to the left of the origin show sessions during which stimulation frequency was lower than the actual peak (established before each session). At least for the most effective protocol (LongCo), greater mismatch is associated with stronger alpha increase.

In addition, we took advantage of the variability of IAF relative to ISF to assess the dependence of α-enhancement on the ISF-to-IAF match in any given session. To this end, we correlated the difference in α-enhancement during tACS relative to sham against the deviation of ISF from actual IAF (i.e. tACS minus sham vs. ISF minus IAF). We found that no active tACS-condition showed stronger α-enhancement with better match between ISF and IAF (Fig. 4). Instead, we found a significant inverse relationship in the most effective condition (LongCo), with stronger tACS-induced α-enhancement for greater deviations between ISF and IAF (Fig. 4, green rectangles, Spearman's *rho* = −0.90, *P* < 0.001). This association remained strong even with the most extreme case removed (Spearman's *rho* = −0.87, *P* < 0.001). A correlation derived from a small sample must be considered with caution but the data show that α-enhancement does not depend on a perfect match between ISF and IAF, contrary to what would be expected from entrainment echoes, and in favor of plasticity effects.

### Analysis of offline α-changes in intermittent, tACS-free intervals

**Figure 5:**
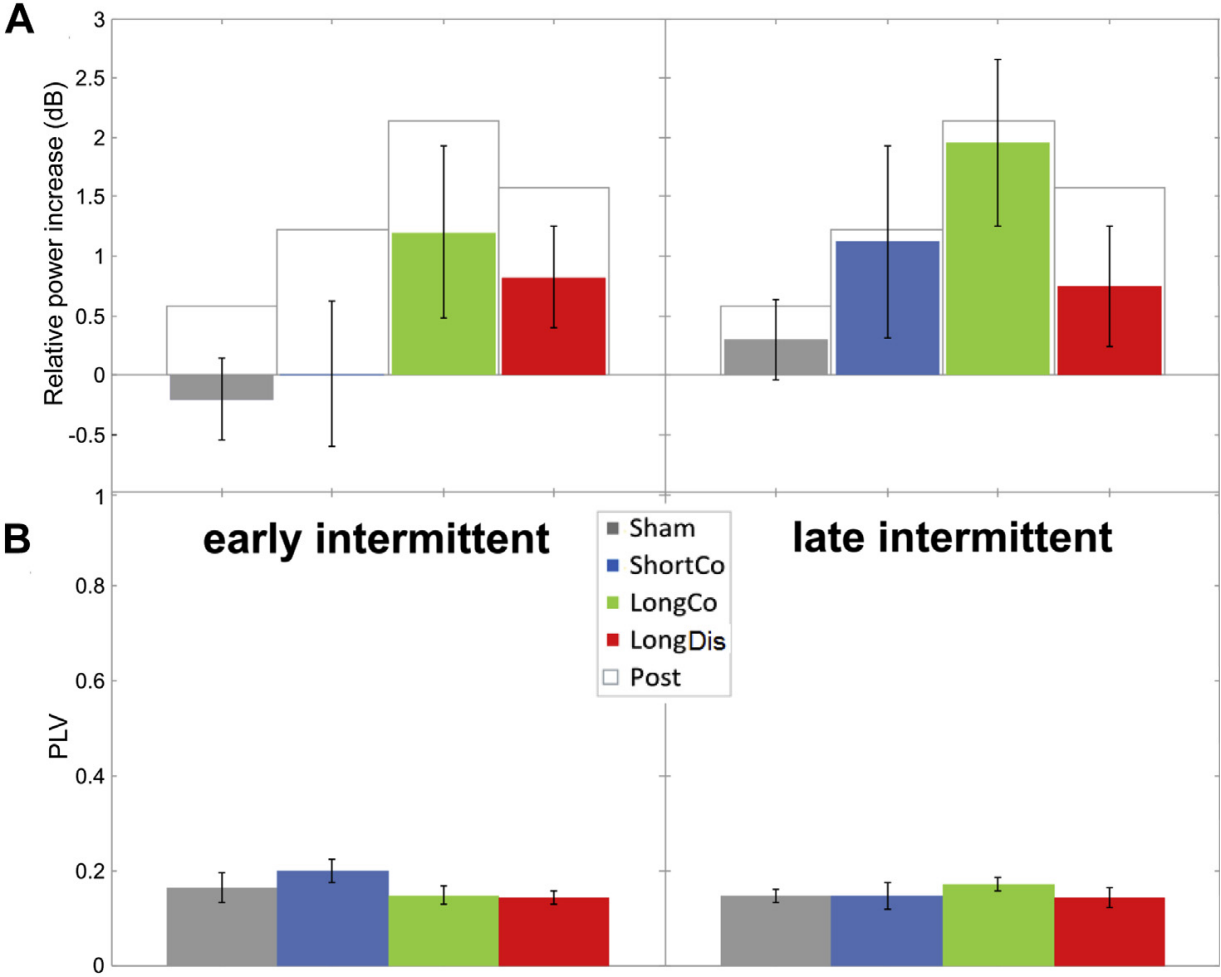
Alpha-effects in intermittent, tACS-free intervals. A) Mean relative increase (dB) in individual alpha band power for early (left) vs late (right) trials during silent periods between stimulation trains compared to pre-test. Grey outline shows mean increase between pre-and post-test for each condition (as shown in Fig. 2A). B) Mean phase locking value across trials for early (left) vs late (right) trials. A value of 0 means no phase locking, a value of 1 means perfect phase locking. There is no evidence for enhanced phase locking. Error bars represent standard error of the mean.

The pattern of tACS-induced α-power changes in the intermittent intervals during stimulation (Fig. 5A) was suggestive of a progressive build-up of the α-aftereffects shown in Fig. 2A (but not significant in either early/late block (*Χ*^2^_3_ = 6.0/4.7, *P* = 0.112/0.195)). Critically, we found no evidence of induced phase-locking (versus sham) in these intervals (i.e. after ∼3 or ∼8 s stimulation with individual tACS trains; Fig. 5B) (early: *Χ*^2^_3_ = 2.5, *P* = 0.48; late *Χ*^2^_3_ = 0.7, *P* = 0.87), again disagreeing with entrainment echoes contributing to the tACS-aftereffects. The absence of phase-locking immediately after tACS-offset shows that online entrainment (if present) does not outlast the tACS trains even between individual trials, and rules out the survival of entrainment echoes for several minutes.

## Discussion

This study tested in a novel intermittent tACS paradigm whether plasticity mechanisms are sufficient to explain α-aftereffects in response to α-tACS. To this end, we manipulated phase continuity and train duration in three discontinuous tACS-protocols with constant total stimulation time and compared tACS-induced offline α-changes against sham. While the previously reported offline α-enhancement [20,24,44,53] was replicated, our data rule out entrainment echoes as a possible explanation of the α-aftereffect in our intermittent protocol, and support the plasticity model as the underlying mechanism. Despite growing evidence for entrainment *during* tACS [20,24,25,30,32,37], our findings indicate that online tACS-entrainment effects may not be strong enough to outlast stimulation, while offline tACS plasticity effects may be present in the absence of entrainment echoes. A similar distinction between online and offline effects has been made for transcranial magnetic stimulation (TMS): Short bursts of rhythmic TMS enhance brain oscillations at TMS-frequency during (i.e. online to) TMS by immediate entrainment [54–56], but prolonged TMS leads to longer-lasting effects on brain oscillations that have been attributed to other mechanisms (i.e. long term potentiation or depression) ([43,57], see also Ref. [58]). An open question is to what extent online entrainment effects and offline plasticity effects are independent. Below we discuss, in light of our and related recent findings, two plasticity models, which assume dependence versus independence of online entrainment and offline plasticity effects, respectively.

### tACS-induced plasticity: the spike-timing dependent plasticity account

**Figure 6:**
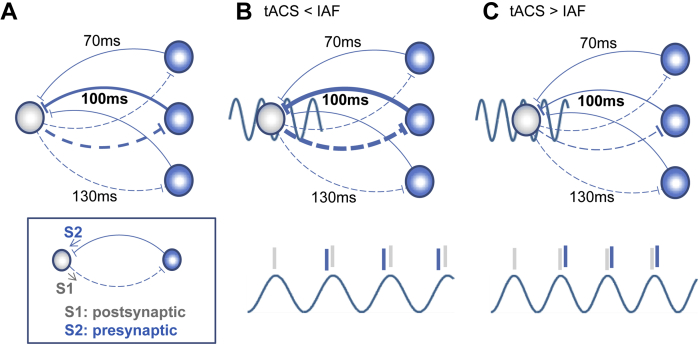
Simplified STDP model of alpha aftereffects by tACS (adapted from Ref. [44]). A) A population of neurons oscillating at alpha-frequencies. Recurrent loops within this population reverberate at different delays, leading to a net oscillatory frequency depending on which connection dominates. Dominant frequency can slowly fluctuate over time/days. In this example, delays of 100 ms dominate, leading to a dominant 10 Hz oscillation. B) and C). Stimulation by tACS. Some neurons are modulated by tACS (grey circles) while others are not (blue circles) (tACS effect is unlikely homogenous across neuronal tissues and locations). Consider the synapse on the grey neurons. Events are triggered rhythmically by tACS (post-synaptic S1, assuming action potential generation shaped by stochastic resonance [87]). These events are then followed by pre-synaptic events (S2) generated through recurrent loops at the delay of the dominant cycle (here 100 ms). When neurons are stimulated at a frequency slightly slower than the dominant frequency of the loop (IAF) (B), pre-synaptic events slightly precede post-synaptic events of the next cycle, leading to strengthening of the synapse (LTP). Conversely, when neurons are stimulated at a frequency slightly faster than the dominant frequency (C), pre-synaptic events slightly follow post-synaptic events of the next cycle, leading to weakening of the synapse (LTD). Note that this model is speculative. While taking into account preferred frequency, and accounting for our data (B), it requires testing for situation (C).

As introduced above, one mechanism that has been proposed to explain tACS-induced α-aftereffects [28,44] is spike-timing dependent plasticity (STDP). In STDP, the order and timing of pre- and post-synaptic potentials determine the magnitude, and direction, of changes in synaptic strength [59–61]. Zaehle et al. [44] used a neural network model incorporating STDP rules to show that periodic 10 Hz-stimulation can strengthen or weaken the synaptic weights of neuronal circuits (recurrent loops) depending on their reverberation frequency. In this model, online entrainment is the window into longer-lasting synaptic plasticity effects that translate into frequency-specific changes in oscillatory activity. The model is illustrated in Fig. 6, which is adapted from [44] with a slight modification: We assume higher weights for selective circuits (here with a periodicity of 100 ms or 10 Hz, see Fig. 6A) to accommodate physiological constraints (here the presence of an individual's dominant α-frequency). This slightly deviates from the model of Zaehle et al. [44], which presumes uniform distribution of starting weights across all loops, i.e. does not explicitly take into account the existence of intrinsic resonance frequencies (although motivated by them). Specifically, with this new assumption, the model predicts synaptic strengthening in dominant (α-) loops when the stimulation frequency falls into a narrow range of frequencies slightly lower than the spontaneous α-peak (Fig. 6B), which is in line with our present results. Under these conditions, post-synaptic events (S1, see Fig. 6A) driven by tACS are generated at a slightly slower pace (<IAF) than the time required for the feedback through the recurrent dominant (α-) loops (resonating at IAF). As a consequence, pre-synaptic (feedback) events (S2, see Fig. 6A) have a higher likelihood to slightly precede the post-synaptic (tACS) events in these loops (see Fig. 6B, bottom), leading to strengthening of their associated synapses. We emphasize that this model is based on a number of assumptions (see also [44]), including that 10 Hz spike bursts result from a 10 Hz alternating current, and that the synaptic strengthening of the effective recurrent loops leads to an increase in natural α-activity. If these assumptions hold, this model matches our data, which show that slower stimulation (relative to IAF) enhances oscillations in the individual α – (here: faster) frequency.

It is important to note that assuming higher weights predicts greater effects at the resonance frequency of a person's dominant circuit when stimulated at nearby frequencies, but lesser or no effects at non-dominant frequencies. In other words, in a participant with a 10 Hz α-peak, aftereffects would predominantly be observed at this intrinsic 10 Hz frequency after stimulation at a nearby frequency (∼10 Hz), but no aftereffects should be observed at non-intrinsic frequencies (e.g. 7 Hz) with stimulation near these frequencies (∼7 Hz) (nor should there be 10 Hz aftereffects after 7 Hz stimulation). In addition, we point out that the assumption of higher weights for dominant oscillations also adds a factor of state-dependency to the model, which is in line with observations from NIBS studies using tACS [17,53,62,63], transcranial random noise stimulation (tRNS) [64], transcranial direct current stimulation (tDCS) [63,65–68], and TMS [69,70], showing that the stimulation outcome is often dependent on the concurrent brain state or the task being executed.

Importantly, this model not only predicts α-enhancement, but also α-suppression as a consequence of synaptic weakening in dominant α-loops when stimulation is applied at slightly faster frequencies relative to the spontaneous α-peak frequency (Fig. 6C). This parallels classical STDP models in which synapses are strengthened when the post-synaptic potential (here: spiking of the driving neuron at tACS-frequency) follows the pre-synaptic potential (here: the feedback to the driving neuron via the recurrent loop), and weakened when the order is reversed. This prediction needs to be verified experimentally.

### tACS-induced plasticity: patterned brain stimulation inducing long-term potentiation or depression

Long-term plasticity and associated effects on brain oscillations have been observed without fine-tuning the stimulation frequency to specific neuronal circuits. For instance, prolonged tDCS, which has no oscillatory component and whose effects have been associated with changes in excitability and synaptic efficacy [71–74], may also lead to enhanced α-activity [75–77]. Hence, other mechanisms than long-lasting STDP in specific reverberating circuits could explain the tACS-aftereffects observed here. For instance, aftereffects of both TMS and tDCS have been related to long term depression (LTD) and potentiation (LTP) [11,12,14,38,72,78,79] depending on parameters which do not show any obvious link to intrinsic brain oscillations. These effects often manifest in cortical excitability changes. As posterior α-activity is taken to be an indicator of cortical excitability [80–82], offline α-changes could reflect these forms of LTD and LTP (but see Ref. [58]). In addition, it should be noted that overall metabolic or perfusion changes might be correlated with, and could possibly explain, excitability/α-changes [83–85]. Predictions derived from such periodicity-independent mechanisms would differ from the STDP account. Unlike with STDP, LTD or LTP should then occur to a similar extent for a broad range of stimulation protocols, such as reported for instance with repetitive TMS where LTD is associated with continuous low-frequency stimulation up to 1 Hz and LTP with interleaved or patterned high-frequency stimulation across many frequencies (5–20 Hz and iTBS) [86]. While our data provide evidence for the plasticity account, it cannot disambiguate between the above SDTP model and alternative mechanisms. Both computational and empirical research is needed to establish the existence and width of specific tACS-frequency windows that give rise to aftereffects, and their relation to intrinsic brain oscillations.

### Limitations of our study

Firstly, our design did not entail a condition with continuous stimulation, precluding a direct comparison between continuous and intermittent tACS aftereffects. It is therefore conceivable that continuous, but not intermittent, tACS leads to lasting entrainment given that in a typical tACS-protocol the brain oscillators are subjected to prolonged phase alignment over thousands of cycles. However, oscillatory phase in EEG recordings is generally instable over time, and as our data show, does not outlive tACS offset for more than 100 ms, thus strengthening our conclusion that the aftereffect is predominantly a consequence of plastic changes. Secondly, we have no information about processes online to tACS. Nonetheless, there is growing evidence that entrainment during tACS is likely, and may even be a prerequisite (though not the underlying process) for plasticity effects (see models above). In line with this view, Helfrich et al. [20] found that participants with greater α-power during tACS – i.e. stronger entrainment – also tended to show greater aftereffects. Thirdly, as in previous studies [20,24,44,53] comparisons to control frequencies are missing. Accordingly, it is unclear how frequency-specific the aftereffects are, although some insight on frequency-specificity can be derived from the observed variability in individual α-frequency with respect to a constant tACS frequency, with aftereffect magnitude being relatively unaffected by frequency mismatch. However, here the size of the mismatch was overall relatively small, and future studies need to clarify whether deviations (small or large) make a difference to outcomes. Moreover, we stimulated below, rather than above IAF. In the light of the STDP model, it will be interesting to determine if the direction of a (small) mismatch has a qualitative influence on the direction of the induced changes. Lastly, there is no data available whether the observed quantitative change in α-power has any functional significance. This needs to be tested through additional behavioral manipulations allowing comparison of performance before and after tACS.

## Conclusion

Offline α-enhancement after α-tACS reflects short-term neural plasticity rather than entrained activity, although it is likely that mechanisms set in motion by online entrainment are prerequisite to such effects. This underlines the potential of tACS as a therapeutic tool. In addition, our findings may be informative for study designs. Given that α-aftereffects were negligible with short trains (3 s) and participants overall tolerated the discontinuous stimulation well, intermittent event-related tACS paradigms with short trains could be viable tools in cognitive research on online tACS effects when potential confounds from aftereffects must be minimized.
