# Supplementary material for: Alpha Power Increase After Transcranial Alternating Current Stimulation at Alpha Frequency (α-tACS) Reflects Plastic Changes Rather Than Entrainment
Source: Brain Stimul. 2015 May-Jun;8(3):499–508. doi: 10.1016/j.brs.2014.12.004 (PMC4464304; doi:10.1016/j.brs.2014.12.004)
Supplement: Supplemental Material and Supplemental Figure S1 [file mmc1.docx]

**Alpha power increase after transcranial alternating current stimulation at alpha-frequency (α-tACS) reflects plastic changes rather than entrainment**

**(Supplementary methods)**

Alexandra Vossen^a^, Joachim Gross^b^, Gregor Thut^b^

^a^School of Psychology & ^b^Institute of Neuroscience and Psychology

University of Glasgow

58 Hillhead Street

Glasgow G12 8QB

United Kingdom

[a.vossen.1@research.gla.ac.uk](mailto:a.vossen.1@research.gla.ac.uk) (A. Vossen)

[Joachim.Gross@glasgow.ac.uk](mailto:Joachim.Gross@glasgow.ac.uk) (J. Gross)

[Gregor.Thut@glasgow.ac.uk](mailto:Gregor.Thut@glasgow.ac.uk) (G. Thut)

Corresponding authors: A. Vossen (Tel: +44 (0)141 330 5151), G. Thut (Tel: +44(0)141 330 3395)

**Supplementary Methods**

***Staircase procedure to determine individual tACS intensity***

Intensity was determined by administering 80 tACS cycles at ISF with increasing intensity from .75mA peak-to-peak (pp) (at which all volunteers reported no or very weak sensations) in steps of .25mA up to 2mA/pp (maximum current density .002857mA/cm^2^, [1]) or until the person reported phosphenes or perceived the stimulation as too uncomfortable. In this case intensity was decreased by .1mA/pp until no phosphenes were detected and until the stimulation was acceptable to the participant.

***Visual Change Detection Task***

To ensure that participants remain vigilant they performed a slow change detection task of the same duration as the stimulation protocol. Stimuli were presented at low frequency and low saliency to minimise interference of visual processing with induced alpha activity. Stimuli were also temporally uncorrelated with tACS on/off-periods. Volunteers were asked to maintain fixation on a white cross centrally presented on a grey background. A red circle of similar luminance as the background (diameter 30pxl) was presented in the lower central visual field (Presentation software, version 16.3, Neurobehavioral Systems, Albany, US). Participants were asked to respond to a colour change from red to green (duration 150ms) by mouse click as quickly as possible. Target events occurred after intervals of between 2.5-4.5min length. After each third of trials a break of 45s was inserted to allow participants to move and blink. While the stimulation protocol was continued during this pause, these trials, and trials containing a target event, were not included in the analysis.

***Choice of non-parametric statistics***

While the increase data were fairly normally distributed, there were two outliers in the LongDis condition and one in the sham condition (criterion: greater or smaller than 1.5 times the interquartile range), two of which remained outliers when ignoring stimulation condition (see Supplemental Fig. S1, left part). The data points moreover belonged to three different participants. In addition, verifying non-normality (for instance by using Kolmogorov-Smirnov or Shapiro-Wilk’s W tests for normality) in a small sample is not very reliable because of low power, which is why non-parametric test are usually considered the “safer” option in small samples. We therefore used non-parametric tests, which do not require that distributions meet stringent criteria.


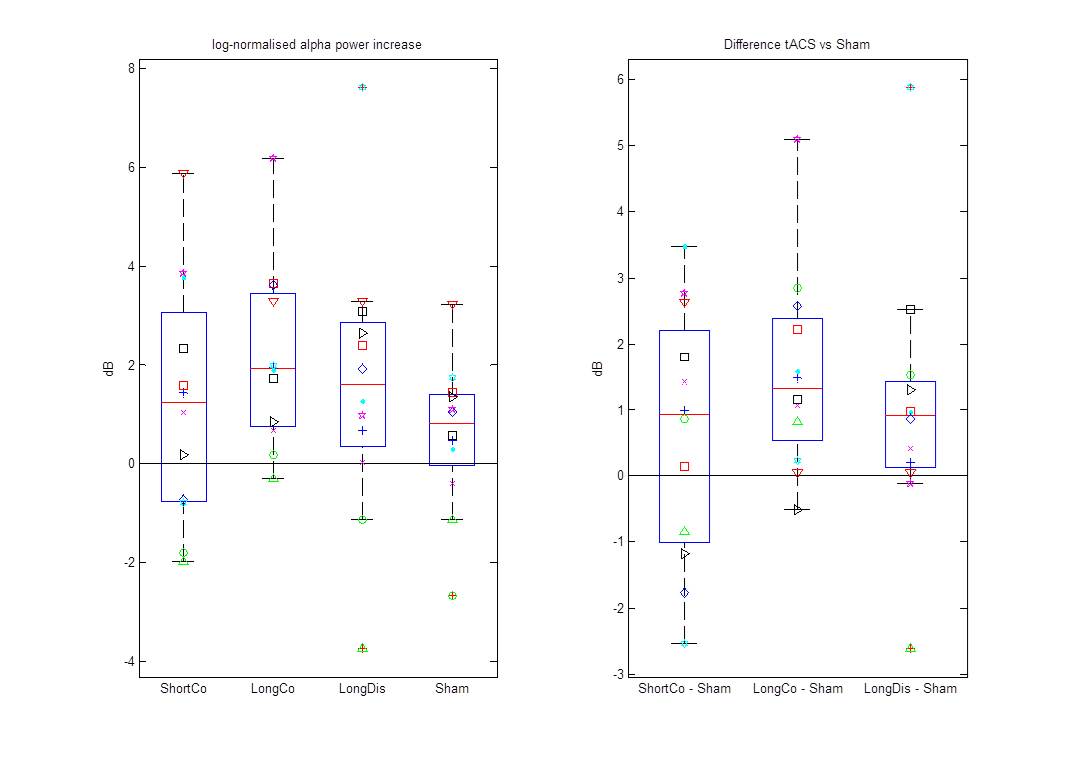


*Supplemental Figure S1.* **Individual results.** *Left:* Alpha increase from pre- to post-test in dB per participant. Each participant is represented by the same symbol in all conditions. Outliers are marked with a red plus sign, horizontal line at zero dB reflects no change. *Right:* Difference score (alpha increase in active condition minus increase after sham) in dB. Note that most participants showed relative greater alpha activity post stimulation in both long (LongCo, LongDis) conditions compared to sham.
